# Biomechanical Evaluation of Ultra-Short Posterior Implants as Adjuncts to Two-Implant Mandibular Overdentures in the Atrophic Edentulous Mandible: A Three-Dimensional Finite Element Analysis Study

**DOI:** 10.3390/jfb17070311

**Published:** 2026-06-24

**Authors:** Ata Mert Yaşa, Ceyda Özçakır Tomruk

**Affiliations:** Department of Oral and Maxillofacial Surgery, Faculty of Dentistry, Yeditepe University, Istanbul 34755, Türkiye

**Keywords:** dental implants, finite element analysis, mandible, overdentures, stress distribution, biomechanics, titanium, alveolar bone loss

## Abstract

Progressive alveolar resorption complicates prosthetic rehabilitation of the atrophic edentulous mandible, and the conventional two-implant mandibular overdenture concentrates occlusal forces on anterior fixtures through extended posterior cantilevers. This study evaluated and compared the biomechanical performance of a conventional two-implant-supported mandibular overdenture with configurations incorporating additional ultra-short (4 mm) posterior implants at varying positions using three-dimensional finite element analysis. A 3D atrophic edentulous mandible model was generated from Visible Human Project computed tomography data. Six configurations were analyzed: Model 1 (control; two 4.1 × 12 mm Ti-15Zr implants at 33 and 43) and Models 2–6, which supplemented the control with two 4.1 × 4 mm ultra-short Ti-15Zr implants at 35–45, 36–46, 35–46, 35–47, and 36–47, respectively. All models were subjected to bilateral oblique (100 N at 45°) and vertical (200 N, 300 N) loading. Von Mises stress, maximum principal stress (P1), and minimum principal stress (P3) were evaluated on all implants. All experimental configurations reduced anterior implant stress compared with the control. Models 5 (35–47) and 4 (35–46) achieved the greatest anterior stress reduction, while Model 6 (36–47) exhibited the most favorable overall stress distribution, with the lowest posterior peak von Mises stress (14.49 MPa). Oblique loading produced higher anterior stress than vertical loading across all models despite its lower magnitude. All stresses remained below the yield strength of Ti-15Zr. Ultra-short posterior implants reduced anterior implant stress and improved load distribution. Asymmetric configurations with a wider anteroposterior spread demonstrated the most favorable biomechanical profiles, supporting their use as minimally invasive adjuncts to standard overdenture protocols.

## 1. Introduction

The atrophic edentulous mandible presents a significant clinical challenge in implant dentistry. Progressive alveolar bone resorption following tooth loss diminishes bone volume and alters the biomechanical environment, complicating prosthetic rehabilitation [1]. As Frost articulated through Wolff’s law, bone remodels in response to the mechanical loads placed upon it; when occlusal stimulation is lost, osteoclast-mediated resorption predominates, leading to progressive atrophy [2]. This relationship between mechanical loading and bone homeostasis underscores the importance of ensuring that implant-supported prostheses transmit forces within physiological limits to preserve peri-implant bone integrity.

The two-implant-supported overdenture, with implants placed in the interforaminal region of the anterior mandible, is a well-established treatment modality that improves denture stability, masticatory function, and patient quality of life [3,4,5]. The McGill and York consensus statements recognized this configuration as the minimum standard of care for edentulous patients [4]. However, this approach often necessitates extended cantilever segments in the posterior denture, concentrating occlusal forces on the anterior implants and surrounding bone [6,7].

Recent advances have explored the use of short (≤8 mm), extra-short (≤6 mm), and ultra-short (≤4 mm) implants in regions of limited vertical bone height, offering a potential alternative to extensive bone augmentation procedures such as onlay grafting, distraction osteogenesis, or nerve lateralization [8,9,10]. Clinical evidence suggests that 4 mm ultra-short implants can achieve predictable outcomes in severely resorbed posterior mandibles, with survival rates comparable to standard-length implants even in atrophic jaws [9,11]; biomechanical finite element analysis (FEA) studies have likewise explored their use in fixed full-arch designs [8].

From a biomechanical perspective, several FEA studies have demonstrated that additional posterior implant support can reduce stress concentrations on anterior fixtures and improve overall load distribution in mandibular overdentures. Alvarez-Arenal et al. compared mandibular two-implant overdentures with implants placed at the lateral incisor, canine, second premolar, or in a crossed anterior–posterior arrangement under both unilateral and bilateral occlusal loading, and reported that the second-premolar configuration yielded the most favorable peri-implant bone stress distribution, while the canine position used in conventional interforaminal overdentures produced higher peri-implant stress, particularly under unilateral posterior load [6]. Şentürk and Akaltan compared the All-on-4 concept with alternative designs and found that models with additional posterior implants had the most favorable stress profiles [12]. Memari et al. compared short (6 mm) and standard (10 mm) implants in mandibular two-implant overdentures and observed comparable stress distribution between the two configurations [13].

Despite these advances, two unresolved questions remain in the literature. First, most biomechanical studies of supplementary ultra-short posterior implants have evaluated only symmetric bilateral arrangements, leaving asymmetric configurations, clinically relevant when bone availability or anatomical landmarks differ between sides, largely uncharacterized. Second, direct comparison against a true two-implant overdenture controlled under a standardized loading scheme is rare [11,14]. The present study addresses both gaps by comparing five posterior implant positional configurations, encompassing both symmetric and asymmetric arrangements, against a true two-implant baseline using a single anatomical model and a standardized force-application area; this design isolates the biomechanical effect of posterior implant position from confounding cantilever-length effects.

The aim of this study was to evaluate and compare the biomechanical behavior of six implant-supported overdenture configurations in an atrophic mandibular model using three-dimensional FEA. Specifically, we sought to determine whether the addition of ultra-short posterior implants at different mandibular positions provides measurable biomechanical advantages in terms of stress distribution across both anterior and posterior implants under clinically relevant loading conditions. The null hypothesis was that the addition of posterior ultra-short implants would not substantially alter the stress distribution pattern compared with the conventional two-implant configuration.

## 2. Materials and Methods

### 2.1. Model Construction

This study was conducted in collaboration between Yeditepe University Faculty of Dentistry and Tinus Technologies. All modeling and analyses were performed on HP workstations (HP Inc., Palo Alto, CA, USA) equipped with Intel Xeon E-2286 processors (2.40 GHz, 64 GB ECC memory, Intel Corporation, Santa Clara, CA, USA).

The mandibular bone model was generated from the Visible Human Project computed tomography (CT) data (National Library of Medicine, Bethesda, MD, USA). Tomography data were reconstructed with 0.33 mm section thickness and segmented in 3D Slicer software (Version 5.6.1; open-source, Brigham and Women’s Hospital and contributors) using appropriate Hounsfield unit thresholds (426.50–3193.04 HU). Three-dimensional models were exported in STL format and refined in Blender software (Version 4.0; Blender Foundation, Amsterdam, The Netherlands). A 0.5 mm inward offset was applied to generate the cortical bone layer, with trabecular bone derived from the inner cortical surface. Oral mucosa was modeled as a 1.8 mm outward offset from the cortical bone surface, and the buccolingual alveolar crest width was set at 5.5 mm.

### 2.2. Implant and Prosthetic Components

The control configuration included two 4.1 × 12 mm Straumann Standard Tissue Level implants (Roxolid^®^, SLActive^®^ surface; Institut Straumann AG, Basel, Switzerland) at positions 33 and 43, according to the Federation Dentaire Internationale (FDI) numbering system. Experimental configurations incorporated two additional 4.1 × 4 mm Straumann Standard Plus Short Tissue Level implants (Roxolid^®^, SLActive^®^ surface; Institut Straumann AG, Basel, Switzerland) at varying posterior positions. In keeping with the convention adopted in the dental implant literature for fixtures with an intra-osseous length of ≤4 mm, these 4 mm fixtures are referred to throughout the present manuscript as ultra-short implants and are distinguished from extra-short (≤6 mm) and short (≤8 mm) categories described elsewhere in the literature [9,11]. Roxolid^®^ is a titanium-zirconium alloy, nominally 83–87 wt% Ti, 13–17 wt% Zr, developed for enhanced mechanical performance relative to commercially pure titanium. All implant components, including Locator^®^ abutments, retention inserts, and metal housings (Zest Dental Solutions, Carlsbad, CA, USA), were modeled to manufacturer catalog dimensions. The six model configurations are described in Table 1.

The polymethyl methacrylate (PMMA) overdenture and acrylic resin teeth were modeled using Wheeler atlas data. All overdenture models extended to the second molar region. Importantly, mastication forces were applied to the same limited area (first premolar, second premolar, and first molar) in each model regardless of the underlying implant configuration, thereby eliminating the potential confounding effect of varying cantilever lengths on the results. A 1 mm thick plate was positioned over these teeth to simulate the occlusal contact area.

To ensure standardization, the ultra-short implants positioned at 35, 36, 45, 46, and 47 were aligned on the same vertical axis. These implants were placed 2.5 mm more apically relative to the control group implants, measured from the standard implant neck regions to the ultra-short implant neck regions.

### 2.3. Mesh Generation and Material Properties

Mathematical models were generated in Altair HyperMesh software (Version 2023; Altair Engineering, Troy, MI, USA) using fine triangular surface meshes (0.1–0.25 mm) and tetrahedral solid elements. The quantitative details of the mesh structures are presented in Table 2. The surface and solid element sizes used in the present analysis lie within the range previously reported to achieve numerical convergence in three-dimensional finite element models of dental implant–bone systems [15,16]; the element-count differences observed across the six configurations, Table 2, reflect the additional geometric complexity introduced by the supplementary ultra-short implants and their peri-implant volumes rather than under-resolution of the underlying meshes, with each model meshed at the same local element-edge length criterion.

The compatibility of force transmission among the models was ensured by adjusting the mesh structures in Altair HyperMesh. All materials were assumed to be homogeneous, isotropic, and linearly elastic. Material properties assigned to each component are summarized in Table 3.

### 2.4. Loading and Boundary Conditions

**Figure 1:**
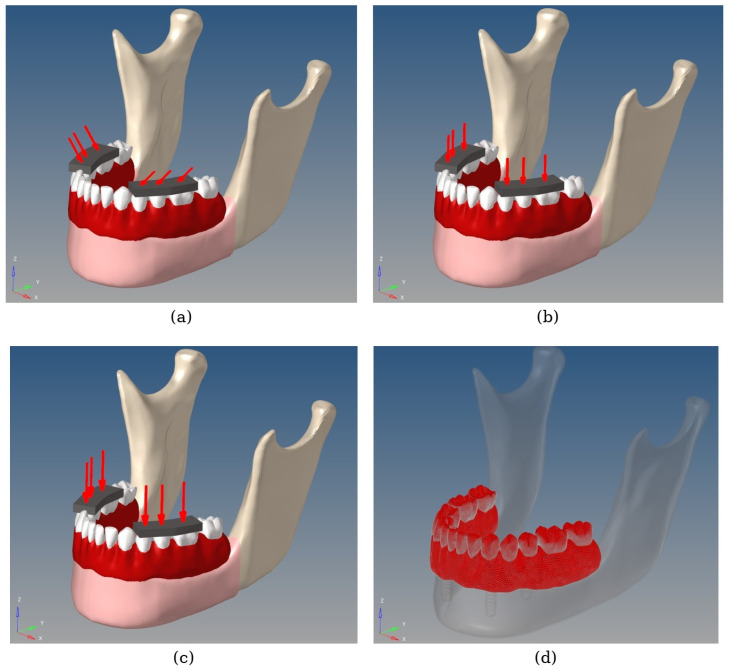
Loading scenarios applied in the finite element analysis: (**a**) bilateral oblique force of 100 N applied at 45° to the buccal aspect; (**b**) bilateral vertical force of 200 N; (**c**) bilateral vertical force of 300 N; (**d**) mesh structure of the overdenture (red).

Three bilateral loading scenarios were applied through the occlusal plate to simulate masticatory function, oblique force of 100 N at 45° to the buccal aspect of the first premolar, second premolar, and first molar regions; vertical force of 200 N applied to the same regions; and vertical force of 300 N applied to the same regions. Forces were distributed across surrounding nodes in the loading regions to avoid stress singularities (Figure 1).

The three loading conditions were chosen to span the range of forces clinically relevant to edentulous overdenture rehabilitation. The oblique 100 N force at 45° simulates the lateral/excursive component of physiological mastication; non-axial forces of this magnitude are biomechanically informative because they generate disproportionately high implant-neck stress through bending-moment amplification, particularly for ultra-short implants [6,12]. The vertical 200 N load corresponds to routine masticatory force commonly applied in mandibular overdenture finite element studies [6,12]. The vertical 300 N load represents a higher-end masticatory force, providing a clinical safety margin while remaining within physiologically realistic values for the target patient population; the patients most commonly indicated for atrophic-mandible overdenture rehabilitation are elderly edentulous individuals, in whom maximum voluntary bite force is substantially reduced relative to dentate counterparts due to the combined effects of edentulism, age-related muscle atrophy, and reduced occlusal support [23]. Jansen van Vuuren et al. reported mean maximum voluntary bite forces of 469.9 N (SD 28.40) for males and 384.5 N (SD 25.3) for females in dentate populations [24], values not realistically attained by the elderly edentulous patient population this model represents. Together, the three scenarios span routine functional to higher-end clinical loading conditions without exceeding physiologically realistic values for the target patient cohort.

**Figure 2:**
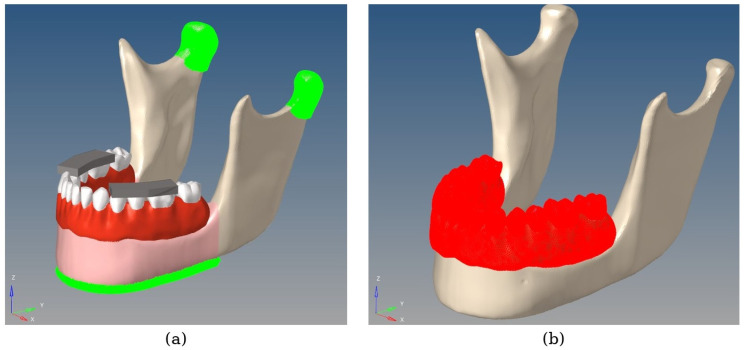
Boundary conditions applied to the finite element model: (**a**) fixed constraints (green) applied at the bilateral condylar regions and along the inferior border of the mandible, with all degrees of freedom restricted in three axes to simulate the physiological stability of the mandible during mastication; (**b**) prosthesis and associated components (red).

All models were constrained at the bilateral condylar regions by fixing all degrees of freedom in three axes. A FREEZE-type contact definition was implemented between all contacting components, assuming complete osseointegration and correlated motion between contacting surfaces. A total of 18 linear static analyses were performed (six models x three loading conditions). Models were solved using the Altair OptiStruct solver (Altair Engineering, Troy, MI, USA) (Figure 2).

### 2.5. Outcome Measures

Three stress parameters were evaluated for all implants in each model: von Mises stress, representing the overall stress state and serving as a predictor of yielding in ductile materials such as titanium alloys; maximum principal stress (P1), indicating the peak tensile stress and zones prone to micro-fracture or bone apposition; and minimum principal stress (P3), representing the peak compressive stress, often associated with cortical bone compression or risk of resorption when excessive [25]. Evaluating these parameters together enables a comprehensive understanding of how different implant configurations distribute both tensile and compressive forces throughout the system [15,16].

### 2.6. Statistical Evaluation

Statistical analysis was not conducted, as the finite element method produces a single deterministic solution for each defined combination of geometry, material properties, and boundary conditions; consequently, repeated analysis of the same model would yield identical numerical output, rendering inferential statistics inapplicable [26]. Comparative interpretation between models was therefore based on direct numerical contrasts of stress magnitudes and distribution patterns, in line with established convention for FEA in implant dentistry [15,16].

## 3. Results

This study evaluated stress distribution under bilateral oblique (100 N) and vertical (200 N, 300 N) loading across six implant configurations using von Mises, P1 major principal (tensile), and P3 minor principal (compressive) stress metrics on both anterior and ultra-short posterior implants.

### 3.1. Anterior Implant Stress

All experimental models (Models 2–6) demonstrated reduced stress values on the anterior implants (33 and 43) compared with the control (Model 1) across all loading conditions and stress metrics. In the symmetrically configured models (Models 1–3), stress values at 33 and 43 were identical, as expected from the bilateral symmetry of these configurations. In asymmetric configurations (Models 4–6), the implant at 33 consistently exhibited lower stress than 43, reflecting the influence of the contralateral posterior implant arrangement.

Oblique loading (100 N) produced the highest von Mises stress values on the anterior implants across all models, ranging from 13.98 MPa (Model 5, 33) to 14.42 MPa (Model 1). Von Mises stress values for all anterior implant positions are presented in Table 4.

The stress reduction on the anterior implants relative to the control, ranged from 0.8% (Model 6) to 7.2% (Model 2) for von Mises stress, from 6.1% (Model 6) to 15.4% (Model 3) for P1 major principal stress, and from 8.1% (Model 6) to 13.5% (Model 5) for P3 minor principal stress. The P1 and P3 values at 33 are presented in Table 5a,b. The corresponding P1 and P3 values at 43 are presented in Table 5a,b.

When anterior implant performance was evaluated by averaging von Mises, P1, and P3 stresses across both implant positions and all loading conditions, Models 5 (35–47) and 4 (35–46) achieved the lowest overall anterior stress values (both at 10.84 MPa, with Model 5 marginally lower), followed by Models 2, 3, and 6. Model 1 (control) exhibited the highest anterior stress (12.01 MPa).

### 3.2. Posterior Implant Stress

The ultra-short posterior implants experienced substantial von Mises stress, particularly under oblique loading. Among the posterior implants, those positioned at 35 (second premolar region, closer to the force application zone) consistently exhibited higher stress than implants at more distal positions such as 46 or 47. The posterior implant stress values for the third and fourth quadrants are presented in Table 6a–c and Table 7a–c, respectively. In configurations with bilaterally symmetric posterior implant arrangements (Models 2 and 3), the ultra-short implants in the third and fourth quadrants experienced identical stress values, consistent with the bilateral symmetry of the model geometry; asymmetric configurations (Models 4, 5, and 6) produced differentiated stress at the two posterior positions.

**Figure 3:**
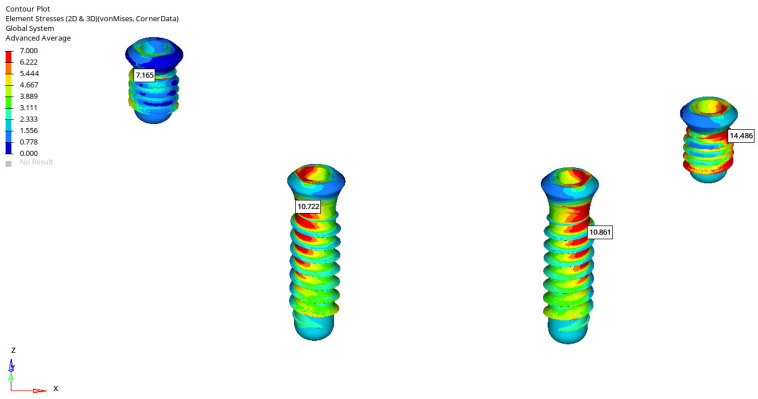
Von Mises stress distribution on implants under vertical 300 N loading (Model 6), showing stress concentration at the implant neck and higher stress on the posterior ultra-short implants.

Model 6 (36–47) demonstrated the most favorable posterior implant stress profile, with an average von Mises stress of 10.34 MPa and the lowest peak von Mises stress of 14.49 MPa. In contrast, Models 2 (35–45), 4 (35–46), and 5 (35–47), all of which included an implant at 35, showed peak posterior von Mises stresses exceeding 20.00 MPa under 300 N vertical loading. Model 3 (36–46) occupied an intermediate position with a peak of 16.17 MPa (Figure 3).

The fourth quadrant implants in Models 5 and 6 (both at 47) showed notably low von Mises stress under vertical loading (4.60 MPa and 4.78 MPa at 200 N, respectively), indicating that the most distal position receives less direct force transmission from the premolar-first molar loading region.

### 3.3. Effect of Loading Direction

**Figure 4:**
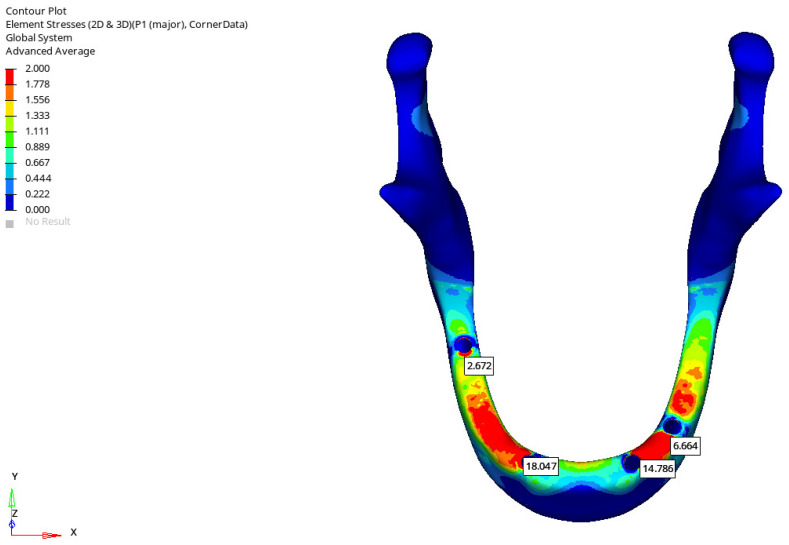
P1 major principal stress (tensile) distribution in the cortical bone under vertical 300 N loading (Model 5), showing elevated tensile stress at the cortical bone interface.

**Figure 5:**
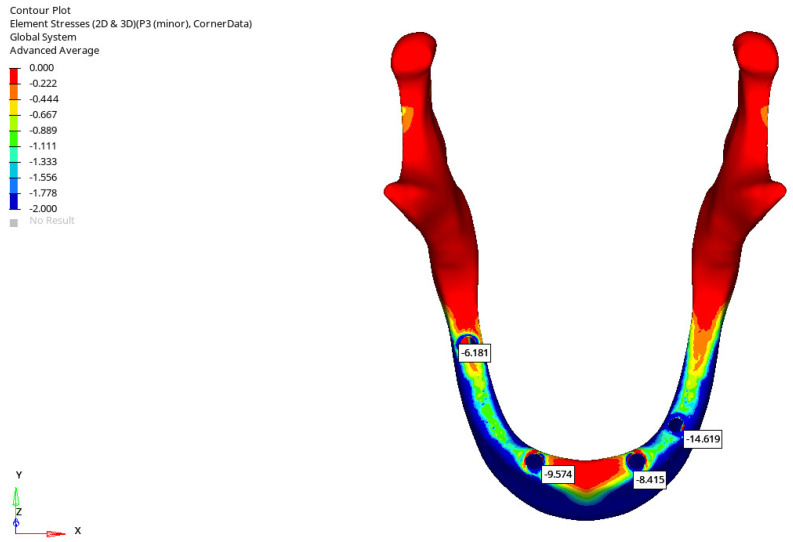
P3 minor principal stress (compressive) distribution in the cortical bone under vertical 300 N loading (Model 5), showing compressive stress concentrated around implant neck regions.

Across all models, oblique loading (100 N) produced higher von Mises stress on the anterior implants than either vertical loading condition (200 N or 300 N), despite the lower applied force magnitude. This finding is consistent with the known amplification of bending moments and crestal stress under non-axial forces. For the posterior ultra-short implants, the 300 N vertical load produced the highest absolute stress values, reflecting the direct compressive load transmission through the overdenture to the shorter posterior fixtures (Figure 4 and Figure 5).

### 3.4. Peri-Implant Bone Stress

In addition to the implant-body stresses reported in Section 3.1 and Section 3.2, peri-implant bone stresses were evaluated separately for the cortical and trabecular compartments at every implant position across all six configurations. Consistent with the convention adopted for non-ductile materials (Section 2.5), maximum principal stress (P1) and minimum principal stress (P3) are reported for the bone domains, whereas von Mises stress was reserved for the metallic implant bodies. As with the implant-body stresses (Section 3.1 and Section 3.2), the bilateral symmetry of Models 1, 2, and 3 produces identical peri-implant bone stress values at the contralateral positions (33 = 43 for the anterior implants; 35 = 45 and 36 = 46 for the posterior implants), whereas the asymmetric configurations (Models 4, 5, and 6) yield differentiated values.

Anterior peri-implant cortical bone stresses (Table 8) followed the same configuration-dependent pattern observed for the anterior implant bodies: the control configuration produced the highest values, and every experimental configuration reduced these values, with Models 5 and 4 yielding the largest reduction at FDI 33 (P1 of 10.09 MPa and 10.43 MPa respectively under oblique 100 N loading, versus 12.51 MPa in the control). The asymmetric models showed differentiated cortical stress at the two anterior positions, with FDI 33 consistently lower than FDI 43, reflecting the proximity of the contralateral posterior implant. Anterior peri-implant trabecular bone stresses are summarized in Table 9.

Trabecular bone stresses at the anterior implants (Table 9) were approximately one to two orders of magnitude lower than the corresponding cortical values across all conditions, consistent with the substantially lower elastic modulus of the cancellous bone modelled (1370 MPa vs. 13,700 MPa for cortical bone). The configuration-dependent pattern paralleled the cortical findings: experimental configurations reduced anterior trabecular bone stress relative to the control, with the asymmetric models showing differentiated values at 33 versus 43. Posterior peri-implant cortical bone stresses at the ultra-short implants are presented in Table 10.

Posterior peri-implant cortical bone stresses (Table 10) mirrored the implant-body pattern reported in Section 3.2: implants positioned at the second premolar site (FDI 35 in Models 2, 4, 5) generated higher cortical P1 stresses (8.17–9.26 MPa under oblique 100 N loading) than implants positioned at the first molar (FDI 36 in Models 3 and 6: 7.26–7.55 MPa) or at the second molar (FDI 47 in Models 5 and 6: 6.87–7.04 MPa). Posterior peri-implant trabecular bone stresses are summarized in Table 11.

In aggregate, the peri-implant bone stress data confirm and extend the implant-body stress findings reported in Section 3.1 and Section 3.2. cortical bone bore the predominant load in every configuration, with trabecular stresses approximately one to two orders of magnitude lower, reflecting the stiffness-dominated load transfer characteristic of the cortical-implant composite. The addition of posterior ultra-short implants reduced peri-implant bone stress at the anterior site in parallel with the reduction in anterior implant-body stress. The most favorable peri-implant bone stress profiles at the posterior implants were obtained in configurations placing the implant at the first or second molar (FDI 36 or 47) rather than at the second premolar (FDI 35).

### 3.5. Comparative Ranking

The performance of the experimental configurations was evaluated from two clinically relevant perspectives: anterior implant protection and overall biomechanical balance.

For anterior implant stress reduction (averaged across von Mises, P1 maximum principal, and the absolute value of P3 minimum principal stress for both FDI 33 and FDI 43 under all loading conditions; absolute values are used because P3 is reported with negative sign as a compressive principal stress, and a signed average would offset the magnitude of compression against the tensile and equivalent stresses rather than combining their magnitudes), the ranking from most to least favorable was: Model 5 ≈ Model 4 > Model 2 > Model 3 > Model 6 > Model 1 (control).

For overall biomechanical performance (mean stress averaged across von Mises, P1, and absolute P3 for every implant in each configuration, under all loading conditions), the ranking from most to least favorable was: Model 6 > Model 5 > Model 3 > Model 4 > Model 2 > Model 1 (control). Model 6 achieved this position primarily due to its substantially lower posterior implant peak stress (14.49 MPa versus >20.00 MPa in Models 2, 4, and 5).

All recorded stress values remained below the yield strength of Ti-15Zr alloy, confirming adequate mechanical safety across all configurations [27].

**Figure 6:**
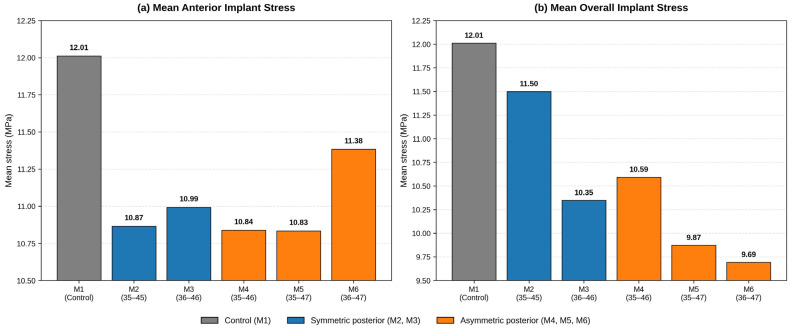
Graphical summary of the dual biomechanical ranking across all six implant configurations: (**a**) mean anterior implant stress (averaged across von Mises, P1, and absolute P3 for both FDI 33 and FDI 43 under all loading conditions), with the control (Model 1, grey) showing the highest values and Models 5 and 4 (asymmetric, orange) showing the lowest; (**b**) mean overall implant stress (averaged across von Mises, P1, and absolute P3 for every implant in each configuration, anterior and posterior, under all loading conditions), with Model 6 showing the lowest mean and the control (Model 1) showing the highest. Bar colors denote configuration type: grey = control, blue = symmetric posterior arrangement, orange = asymmetric posterior arrangement.

A graphical summary of the dual ranking across all six configurations is presented in Figure 6.

## 4. Discussion

This study evaluated the biomechanical effects of supplementing a conventional two-implant mandibular overdenture with additional ultra-short posterior implants at five different positional configurations. The null hypothesis was rejected: the addition of posterior ultra-short implants substantially altered stress distribution patterns compared with the two-implant control, reducing anterior implant stress by 0.8–13.5% depending on the configuration and stress metric evaluated. This finding that all experimental models reduced anterior implant stress aligns with the fundamental biomechanical principle that additional support points distribute occlusal forces more broadly, reducing load concentration on individual fixtures [2].

Improper distribution of stress, whether insufficient or excessive, can cause bone resorption; when physiological limits and thresholds are maintained, a balance between resorptive and appositional activities can be reached, ensuring preservation of bone integrity [2]. This observation is consistent with previous FEA studies demonstrating the benefits of posterior implant support in mandibular overdentures. Daas et al. examined two-implant-retained overdentures and found that rigid attachment systems can lead to localized stress concentration in peri-implant cortical bone, supporting the rationale for additional posterior support [17]. Liu et al. and Cicciù et al. evaluated overdentures supported by four implants and demonstrated significant reductions in peak stresses and more uniform load distribution compared with two-implant designs [28,29]. Şentürk and Akaltan reported that configurations with additional posterior implants or splinted frameworks exhibited the most favorable stress profiles compared with traditional All-on-4 designs [12].

In the control configuration, the absence of posterior implant support means that occlusal forces applied to the loaded premolar-first molar region must be transmitted entirely through the posterior portion of the overdenture base to the anterior interforaminal implants at FDI 33 and 43. This force pathway functions mechanically as an extended posterior cantilever loaded at its distal end, with the anterior implants acting as the fulcrum; the result is a substantial bending moment at the anterior implant necks and a corresponding concentration of compressive and tensile stress in the surrounding peri-implant bone. The addition of posterior ultra-short implants at any of the five experimental positions interrupts this cantilever-mediated pathway by providing direct intermediate support beneath the loaded zone, reducing both the magnitude of force reaching the anterior fixtures and the bending moment they must absorb. This mechanical interruption is the underlying reason that all five experimental configurations reduced anterior implant stress relative to the control, independent of the specific posterior position chosen.

A key finding of this study is that the optimal configuration depends on the clinical priority. When the primary objective is to minimize stress on the anterior implants, which bear the greatest biomechanical responsibility for overdenture retention and have the most critical bone–implant interface, Models 5 (35–47) and 4 (35–46) perform best. Both Models 4 and 5 include an implant at 35, which provides a nearby support point that directly counteracts forces applied to the premolar region. The near-equivalence of these two models, with an average anterior stress difference of only 0.02%, suggests that for anterior protection specifically, the position of the contralateral posterior implant (46 vs. 47) has minimal additional effect.

However, when overall biomechanical balance is considered, including the stress experienced by the ultra-short posterior implants themselves, Model 6 (36–47) emerges as the most favorable configuration. This result is attributable to several converging biomechanical factors that operate at the FDI 35 position. First, FDI 35 sits at the mesial edge of the loaded premolar-first molar zone, placing it within the direct force-application area; the more distal positions (FDI 36, 46, 47) lie progressively farther from the primary load and receive force transmitted through the prosthesis with a longer effective lever arm, attenuating the peak stress reaching each implant. Second, with the anterior implants at FDI 33/43 acting as the primary anterior pivot, FDI 35 functions as the closest posterior support point and bears a disproportionate share of the loaded segment as the prosthesis transmits force forward; this fulcrum effect concentrates stress at the FDI 35 implant neck. Third, the limited bone–implant contact area available at 4 mm fixture length amplifies the per-unit-area stress that develops in response to any given external force, rendering ultra-short implants inherently more susceptible to stress concentration [10,11]. For instance, under 300 N vertical loading, the third quadrant implant at 35 reached von Mises stress values exceeding 20.00 MPa in Models 2, 4, and 5, whereas the corresponding implant at 36 in Models 3 and 6 remained below 15.00 MPa, illustrating that even a single tooth position more distally moves the implant out of the primary loading zone and substantially reduces peak stress. Minimizing peak stress on these fixtures is clinically relevant for long-term implant survival. This finding aligns with Schimmel et al., who demonstrated acceptable clinical outcomes for ultra-short implants in mandibular fixed prostheses [30], and with Arroyo et al., who reported successful immediate loading of short implants in atrophic mandibles [31].

From a clinical practicability standpoint, asymmetric ultra-short posterior implant arrangements are not an a priori design choice but rather a configuration that becomes appropriate when bilateral symmetric placement is anatomically or biologically constrained. In severely atrophic edentulous mandibles, the available residual bone volume in the posterior region is frequently asymmetric, owing to differential rates of resorption following the extraction sequence, unilateral previous trauma or surgery, asymmetric mental-foramen position, or unilateral pathology such as residual cysts or socket-preservation defects; in these patients a fully symmetric posterior implant scheme may not be feasible without extensive contralateral bone augmentation. The asymmetric configurations evaluated here (Models 4, 5 and 6) represent the practical implant positions a clinician might consider when faced with these anatomical constraints, and demonstrating that such configurations preserve favorable biomechanical behavior is therefore directly clinically relevant. Recognized limitations of asymmetric arrangements in everyday practice include the need for occlusal-scheme adjustments to avoid asymmetric loading at the prosthetic surface, somewhat greater complexity in retentive-element selection and maintenance, and the requirement for individualized stress-bearing planning during prosthesis design. None of these limitations preclude the use of asymmetric configurations in suitably selected patients, but they should be weighed alongside the biomechanical advantages demonstrated in the present FEA.

To comprehensively assess the biomechanical environment around the implants, three distinct stress metrics were analyzed. Von Mises stress serves as a reliable predictor of yielding and potential structural failure, particularly for ductile materials such as titanium alloys [15,16]. P1 major principal stress reflects the maximum tensile stress, indicating zones prone to tensile strain and potential micro-fracture or bone apposition. P3 minor principal stress represents the maximum compressive stress, often associated with cortical bone compression or risk of resorption when excessive [25]. In the present models, stresses were consistently concentrated in the marginal peri-implant region and along the implant threads, in agreement with previous FEA reports on implant-supported overdentures [32,33,34,35].

Numerous finite element studies have shown that peak tensile and compressive principal stresses tend to concentrate in the crestal cortical bone and around the implant neck, with this region consistently exhibiting higher stress levels than the cancellous compartment, particularly in atrophic jaws with reduced cortical thickness [36,37,38,39]. By comparing five distinct posterior implant placements rather than the more common add-or-not-add design, the present analysis provides positional resolution of the stress redistribution within this established literature pattern, rather than re-demonstrating that posterior support reduces anterior loading.

The high elastic modulus of the implant material Ti-15Zr (105,000 MPa) allows it to absorb a significant portion of masticatory forces within the implant body, thereby reducing the stress transferred to the bone. The stiffness disparity between the elastic modulus of the bone and the implant material leads to the concentration of the majority of the load within the implant body [18]. The Ti-15Zr (Roxolid^®^) alloy used in these implants has been mechanically characterised with an ultimate tensile strength of approximately 953 MPa [18], with a static yield strength reported to be 10–15% higher than that of commercially pure Grade IV titanium and a fatigue endurance limit of 500–560 MPa, depending on the surface treatment applied [19]. The maximum stress recorded in the present models (21.35 MPa at the third-quadrant ultra-short implant in Model 2 under 300 N vertical loading) thus corresponds to approximately 2.2% of the tensile strength and 4.3% of the fatigue endurance limit reported for the sand-blasted and acid-etched surface used clinically, indicating substantial safety margins under both static and cyclic loading conditions. Static finite element analysis does not itself capture cumulative cyclic fatigue effects from millions of masticatory cycles; however, long-term implant durability under repeated functional loading remains an important separate consideration further addressed in the limitations [27].

Differences in cortical and trabecular bone thickness, particularly the thin cortical bone, influenced stress distribution patterns across regions. Yang et al. [10] have shown that when cortical bone is thin and when it directly absorbs occlusal deformation, the highest stress concentration in the posterior region shifts to the underlying trabecular bone, producing an inverse relationship between the two bone compartments.

The consistently higher stress observed under oblique loading compared with vertical loading, despite the lower force magnitude (100 N vs. 200–300 N), underscores the importance of occlusal scheme design in implant-supported overdentures. This finding is consistent with previous reports demonstrating that non-axial forces amplify bending moments and crestal bone stress [2,10,40]. Clinically, this suggests that balanced occlusal contacts and avoidance of lateral excursive interferences may be particularly important in overdenture configurations with ultra-short posterior implants.

The clinical contribution of the present analysis is therefore not the existence of this redistribution, which is well established, but the position-resolved quantification that permits clinicians to choose among configurations based on whether anterior protection or overall biomechanical balance is the primary objective. Our findings corroborate previous studies evaluating implant-supported overdentures under varying conditions. Tükel and Küçükkurt evaluated extra-short implants in the posterior mandible as a means of avoiding extensive bone grafting and reported a more risk-free stress distribution under molar-region loading, although their effect on canine-region forces was less pronounced [8]. The results of this study also align with the biomechanical principles outlined by Hu et al., which emphasized the necessity of individualized treatment planning based on bone density and quality [41]. The higher stress concentrations observed at the cortical bone interface are consistent with Arat Bilhan et al., who analyzed different attachment types and implant numbers supporting mandibular overdentures and found that posterior support plays a crucial role in stabilizing occlusal forces [42].

Although direct in vivo strain measurement at the bone–implant interface is not feasible in patients, the present finite element model can be cross-validated against the body of published three-dimensional FEA work on mandibular overdenture configurations. The computed implant-body stress magnitudes (peak von Mises ≈ 21 MPa under 300 N vertical loading) fall within the range reported by prior FEA studies of mandibular two-implant and multi-implant overdentures under comparable loading conditions [6,12,28,29], and the qualitative stress-distribution patterns observed here reproduce findings consistently reported across this literature [6,12,17,18,28,29,32,39]. This convergence with prior independent studies supports the methodological adequacy of the present model for the inter-configuration comparisons drawn in this work, while recognizing that the absence of formal in vitro benchmarking remains a limitation common to FEA studies of implant biomechanics, as discussed in the next subsection.

Several limitations should be acknowledged. First, all materials were modeled as homogeneous, isotropic, and linearly elastic, a standard simplification in dental FEA that aids computational efficiency but reduces biological realism [16]. Second, complete osseointegration was assumed at all implant–bone interfaces through the use of a FREEZE-type contact definition, which represents an idealized upper bound on biomechanical performance. This assumption is particularly consequential for the ultra-short implants, where the quality and extent of osseointegration are among the principal determinants of clinical outcome and where systemic factors such as inflammation, metabolic conditions, and concurrent medication can produce sub-optimal integration. The reported stress magnitudes should therefore be interpreted as a best-case scenario; in clinical practice, partial osseointegration or peri-implant bone remodeling would alter the local stiffness of the implant–bone composite and consequently the stress distribution. Third, the applied loads represented simplified static approximations of complex, dynamic masticatory forces. Implant micromotion and the displacement response under physiological loading, both especially relevant for ultra-short implants where primary stability depends on a limited bone–implant interface, were not analyzed in the present static stress evaluation; future work should include displacement and microstrain analysis to capture these complementary outcomes. Furthermore, the present static finite element analysis does not capture cumulative fatigue effects under cyclic masticatory loading, dental implants are subjected to millions of loading cycles during their service life, and even when peak static stresses remain well below the alloy yield strength, fatigue-related failure modes can develop over time; dynamic loading studies and fatigue-specific analyses would extend the clinical translatability of these findings. Fourth, only a limited number of posterior implant positions were evaluated; additional configurations, including alternative implant positions, designs, and surface treatments, warrant investigation. Fifth, both cortical and trabecular bone layers were modeled as isotropic and homogeneous, despite the known anisotropy of natural bone tissue. Finally, the results are derived from a single standardized mandibular model and do not account for patient-specific anatomical variability; future patient-specific simulations incorporating patient-derived CT scans would allow more personalized biomechanical analyses and could reveal how anatomical variability influences stress distribution. Despite these limitations, the study provides a systematic, controlled comparison of multiple posterior ultra-short implant positions within a consistent modeling framework, an approach that isolates the biomechanical effect of implant position while controlling for all other variables.

## 5. Conclusions

Within the limitations of the present finite element analysis, the addition of ultra-short posterior implants to a conventional two-implant mandibular overdenture reduced anterior implant stress across all tested configurations. Configurations including an implant at the second premolar position (Models 5 and 4) yielded the greatest anterior stress reduction, while Model 6 produced the most favorable overall biomechanical balance by minimizing peak stress on the posterior ultra-short implants. Oblique loading produced higher anterior implant stress than vertical loading despite a lower force magnitude, supporting the relevance of careful occlusal-scheme design. All recorded stresses remained well below the mechanical limits of the Ti-15Zr alloy, under simulated static functional loading. These computational findings highlight that, when clinical indications necessitate, asymmetrically positioned ultra-short implants may serve as a biomechanically rational, minimally invasive adjunct to two-implant overdenture protocols in atrophic edentulous mandibles; however, the observed stress reductions of a few percent to approximately 15% should be interpreted within the static, idealized nature of the present analysis, and longitudinal clinical trials remain necessary to validate these predictions under cyclic functional loading.

## Figures and Tables

**Table 1:** Model configurations.

Model	Configuration
Model 1 (Control (C))	2 Straumann Standard Tissue Level implants (4.1 × 12 mm) at 33, 43 + overdenture
Model 2	(C) + 2 Straumann Standard Plus Short Tissue Level implants (4.1 × 4 mm) at 35, 45 + overdenture
Model 3	(C) + 2 Straumann Standard Plus Short Tissue Level implants (4.1 × 4 mm) at 36, 46 + overdenture
Model 4	(C) + 2 Straumann Standard Plus Short Tissue Level implants (4.1 × 4 mm) at 35, 46 + overdenture
Model 5	(C) + 2 Straumann Standard Plus Short Tissue Level implants (4.1 × 4 mm) at 35, 47 + overdenture
Model 6	(C) + 2 Straumann Standard Plus Short Tissue Level implants (4.1 × 4 mm) at 36, 47 + overdenture

**Table 2:** Quantitative model information.

Model	Total Nodes	Total Elements
Model 1	320,559	1,224,170
Model 2	423,261	1,632,997
Model 3	422,926	1,630,530
Model 4	820,598	3,171,666
Model 5	822,055	3,176,993
Model 6	823,592	3,178,855

**Table 3:** Material properties assigned in the finite element models.

Material	Elastic Modulus (MPa)	Poisson’s Ratio (ν)	Density (kg/m^3^)	Source
Cortical bone	13,700	0.30	1300	[17]
Trabecular bone	1370	0.30	1300	[17]
Ti-15Zr	105,000	0.34	4500	[18,19]
Acrylic resin	2600	0.30	1185	[20]
Mucosa	680	0.45	-	[20,21]
PMMA	2000	0.38	1185	[22]

**Table 4:** Von Mises stress (MPa) at anterior implant positions across all models and loading conditions.

Model	Position	Obl 100 N	V 200 N	V 300 N
1 (Control)	33	14.42	7.25	10.88
1 (Control)	43	14.42	7.25	10.88
2 (35–45)	33	14.07	6.45	9.68
2 (35–45)	43	14.07	6.45	9.68
3 (36–46)	33	14.20	7.00	10.51
3 (36–46)	43	14.20	7.00	10.51
4 (35–46)	33	14.02	6.44	9.66
4 (35–46)	43	14.23	6.82	10.22
5 (35–47)	33	13.98	6.41	9.62
5 (35–47)	43	14.28	6.93	10.40
6 (36–47)	33	14.25	7.24	10.86
6 (36–47)	43	14.33	7.15	10.72

**Table 5:** (**a**) P1 major principal stress and P3 minor principal stress (MPa) at anterior implant position 33 across all models and loading conditions. (**b**) P1 major principal stress and P3 minor principal stress (MPa) at anterior implant position 43 across all models and loading conditions.

(**a**)						
**Model**	**P1 Obl 100 N**	**P1 V 200 N**	**P1 V 300 N**	**P3 Obl 100 N**	**P3 V 200 N**	**P3 V 300 N**
1 (Control)	12.51	12.16	18.24	−14.52	−7.25	−10.87
2 (35–45)	11.69	10.62	15.92	−13.63	−6.29	−9.44
3 (36–46)	12.77	9.40	14.11	−13.82	−6.85	−10.27
4 (35–46)	10.43	10.29	15.44	−13.13	−5.91	−8.68
5 (35–47)	10.09	9.86	14.79	−12.77	−5.61	−8.41
6 (36–47)	12.81	9.80	14.71	−14.53	−5.83	−8.75
(**b**)						
**Model**	**P1 Obl 100 N**	**P1 V 200 N**	**P1 V 300 N**	**P3 Obl 100 N**	**P3 V 200 N**	**P3 V 300 N**
1 (Control)	12.51	12.16	18.24	−14.52	−7.25	−10.87
2 (35–45)	11.69	10.62	15.92	−13.63	−6.29	−9.44
3 (36–46)	12.77	9.40	14.11	−13.82	−6.85	−10.27
4 (35–46)	11.80	11.37	17.05	−13.66	−6.37	−9.56
5 (35–47)	12.15	12.03	18.05	−13.68	−6.38	−9.57
6 (36–47)	13.01	12.10	18.15	−13.67	−6.97	−10.19

**Table 6:** (**a**) Von Mises stress (MPa) at posterior implant positions (third quadrant) across experimental models and loading conditions. (**b**) P1 major principal stress (MPa) at posterior implant positions (third quadrant) across experimental models and loading conditions. (**c**) P3 minor principal stress (MPa) at posterior implant positions (third quadrant) across experimental models and loading conditions.

(**a**)				
**Model**	**Position**	**Obl 100 N**	**V 200 N**	**V 300 N**
2 (35–45)	35	16.29	14.23	21.35
3 (36–46)	36	16.17	9.74	14.61
4 (35–46)	35	16.04	14.02	21.04
5 (35–47)	35	15.97	13.99	20.99
6 (36–47)	36	14.27	9.66	14.49
(**b**)				
**Model**	**Position**	**Obl 100 N**	**V 200 N**	**V 300 N**
2 (35–45)	35	9.26	5.04	7.55
3 (36–46)	36	7.55	3.74	5.61
4 (35–46)	35	8.17	4.93	7.39
5 (35–47)	35	7.86	4.44	6.66
6 (36–47)	36	7.26	3.71	5.57
(**c**)				
**Model**	**Position**	**Obl 100 N**	**V 200 N**	**V 300 N**
2 (35–45)	35	−10.41	−10.03	−15.04
3 (36–46)	36	−9.40	−8.15	−12.22
4 (35–46)	35	−9.55	−9.90	−14.85
5 (35–47)	35	−9.53	−9.75	−14.62
6 (36–47)	36	−8.01	−7.50	−11.24

**Table 7:** (**a**) Von Mises stress (MPa) at posterior implant positions (fourth quadrant) across experimental models and loading conditions. (**b**) P1 major principal stress (MPa) at posterior implant positions (fourth quadrant) across experimental models and loading conditions. (**c**) P3 minor principal stress (MPa) at posterior implant positions (fourth quadrant) across experimental models and loading conditions.

(**a**)				
**Model**	**Position**	**Obl 100 N**	**V 200 N**	**V 300 N**
2 (35–45)	45	16.29	14.23	21.35
3 (36–46)	46	16.17	9.74	14.61
4 (35–46)	46	14.31	8.47	12.71
5 (35–47)	47	11.36	4.60	6.90
6 (36–47)	47	11.65	4.78	7.17
(**b**)				
**Model**	**Position**	**Obl 100 N**	**V 200 N**	**V 300 N**
2 (35–45)	45	9.26	5.04	7.55
3 (36–46)	46	7.55	3.74	5.61
4 (35–46)	46	7.06	4.93	3.77
5 (35–47)	47	6.87	4.44	2.67
6 (36–47)	47	7.04	3.71	5.64
(**c**)				
**Model**	**Position**	**Obl 100 N**	**V 200 N**	**V 300 N**
2 (35–45)	45	−10.41	−10.03	−15.04
3 (36–46)	46	−9.40	−8.42	−12.22
4 (35–46)	46	−9.76	−7.74	−11.61
5 (35–47)	47	−9.47	−4.12	−6.18
6 (36–47)	47	−10.28	−4.81	−7.22

**Table 8:** P1 major principal stress and P3 minor principal stress (MPa) in the peri-implant cortical bone at the anterior interforaminal implants (FDI 33 and 43) across all models and loading conditions.

Model	Position	P1 Obl 100 N	P1 V 200 N	P1 V 300 N	P3 Obl 100 N	P3 V 200 N	P3 V 300 N
1 (control)	33	12.51	12.16	18.24	−14.52	−7.25	−10.87
1 (control)	43	12.51	12.16	18.24	−14.52	−7.25	−10.87
2 (35–45)	33	11.69	10.62	15.92	−13.63	−6.29	−9.44
2 (35–45)	43	11.69	10.62	15.92	−13.63	−6.29	−9.44
3 (36–46)	33	12.77	9.40	14.11	−13.82	−6.85	−10.27
3 (36–46)	43	12.77	9.40	14.11	−13.82	−6.85	−10.27
4 (35–46)	33	10.43	10.29	15.44	−13.13	−5.91	−8.87
4 (35–46)	43	11.80	11.37	17.05	−13.66	−6.37	−9.56
5 (35–47)	33	10.09	9.86	14.79	−12.77	−5.61	−8.41
5 (35–47)	43	12.15	12.03	18.05	−13.68	−6.38	−9.57
6 (36–47)	33	12.81	9.80	14.71	−14.53	−5.83	−8.75
6 (36–47)	43	13.01	12.10	18.15	−13.67	−6.79	−10.19

**Table 9:** P1 major principal stress and P3 minor principal stress (MPa) in the peri-implant trabecular bone at the anterior interforaminal implants (FDI 33 and 43) across all models and loading conditions.

Model	Position	P1 Obl 100 N	P1 V 200 N	P1 V 300 N	P3 Obl 100 N	P3 V 200 N	P3 V 300 N
1 (control)	33	0.24	0.22	0.33	−0.49	−0.41	−0.62
1 (control)	43	0.24	0.22	0.33	−0.49	−0.41	−0.62
2 (35–45)	33	0.23	0.21	0.32	−0.46	−0.37	−0.55
2 (35–45)	43	0.23	0.21	0.32	−0.46	−0.37	−0.55
3 (36–46)	33	0.23	0.19	0.29	−0.47	−0.37	−0.56
3 (36–46)	43	0.23	0.19	0.29	−0.47	−0.37	−0.56
4 (35–46)	33	0.23	0.21	0.32	−0.45	−0.35	−0.53
4 (35–46)	43	0.23	0.22	0.33	−0.46	−0.40	−0.61
5 (35–47)	33	0.22	0.21	0.31	−0.45	−0.34	−0.51
5 (35–47)	43	0.24	0.23	0.34	−0.47	−0.40	−0.60
6 (36–47)	33	0.25	0.20	0.30	−0.47	−0.40	−0.60
6 (36–47)	43	0.26	0.24	0.36	−0.47	−0.41	−0.62

**Table 10:** P1 major principal stress and P3 minor principal stress (MPa) in the peri-implant cortical bone at the third- and fourth-quadrant ultra-short posterior implants across the experimental models and loading conditions.

Model	Position	P1 Obl 100 N	P1 V 200 N	P1 V 300 N	P3 Obl 100 N	P3 V 200 N	P3 V 300 N
2 (35–45)	35	9.26	5.04	7.55	−10.41	−10.03	−15.04
2 (35–45)	45	9.26	5.04	7.55	−10.41	−10.03	−15.04
3 (36–46)	36	7.55	3.74	5.61	−9.40	−8.15	−12.22
3 (36–46)	46	7.55	3.74	5.61	−9.40	−8.15	−12.22
4 (35–46)	35	8.17	4.93	7.39	−9.55	−9.90	−14.85
4 (35–46)	46	7.06	2.51	3.77	−9.76	−7.74	−11.61
5 (35–47)	35	7.86	4.44	6.66	−9.53	−9.75	−14.62
5 (35–47)	47	6.87	1.78	2.67	−9.47	−4.12	−6.18
6 (36–47)	36	7.26	3.71	5.57	−8.01	−7.50	−11.24
6 (36–47)	47	7.04	3.76	5.64	−10.28	−4.81	−7.22

**Table 11:** P1 major principal stress and P3 minor principal stress (MPa) in the peri-implant trabecular bone at the third- and fourth-quadrant ultra-short posterior implants across the experimental models and loading conditions.

Model	Position	P1 Obl 100 N	P1 V 200 N	P1 V 300 N	P3 Obl 100 N	P3 V 200 N	P3 V 300 N
2 (35–45)	35	0.55	2.79	4.19	−1.20	−0.25	−0.38
2 (35–45)	45	0.55	2.79	4.19	−1.20	−0.25	−0.38
3 (36–46)	36	0.41	0.99	1.48	−0.80	−0.23	−0.35
3 (36–46)	46	0.41	0.99	1.48	−0.80	−0.23	−0.35
4 (35–46)	35	0.53	2.27	3.40	−0.96	−0.21	−0.32
4 (35–46)	46	0.26	0.92	1.37	−1.14	−0.24	−0.36
5 (35–47)	35	0.46	2.23	3.35	−0.94	−0.18	−0.26
5 (35–47)	47	0.22	0.76	1.14	−1.15	−0.24	−0.36
6 (36–47)	36	0.44	0.91	1.36	−0.68	−0.16	−0.24
6 (36–47)	47	0.63	0.77	1.15	−0.77	−0.27	−0.41

## Data Availability

The raw data supporting the conclusions of this article will be made available by the authors on reasonable request.

## Abbreviations

The following abbreviations are used in this manuscript:

CT	Computed Tomography
FDI	Fédération Dentaire Internationale
FEA	Finite Element Analysis
HU	Hounsfield Unit
P1	Maximum Principal Stress
P3	Minimum Principal Stress
PMMA	Polymethyl Methacrylate
Ti-15Zr	Titanium-15% Zirconium Alloy
VHP	Visible Human Project
VM	Von Mises (stress)
